# Cancer Stem Cells: Biological Functions and Therapeutically Targeting

**DOI:** 10.3390/ijms15058169

**Published:** 2014-05-09

**Authors:** Marius Eugen Ciurea, Ada Maria Georgescu, Stefana Oana Purcaru, Stefan-Alexandru Artene, Ghazaleh Hooshyar Emami, Mihai Virgil Boldeanu, Daniela Elise Tache, Anica Dricu

**Affiliations:** 1 Faculty of Medicine, University of Medicine and Pharmacy of Craiova, Str. Petru Rares nr. 2-4, Craiova 710204, Romania; E-Mails: meciurea@gmail.com (M.E.C.); ada_georgescu@yahoo.com (A.M.G.); stoapo@yahoo.com (S.O.P.); stefan.artene@yahoo.com (S.-A.A.); ghazaleh_ei@yahoo.co.uk (G.H.E.); dodolita@yahoo.it (D.E.T.); 2 Stem Cell Bank Unit, Medico Science SRL, Str. Brazda lui Novac nr. 1B, Craiova 200690, Romania; E-Mail: info@medicoscience.ro

**Keywords:** cancer stem cell, tumorigenicity, signalling pathways, cancer stem cell markers, cancer therapy

## Abstract

Almost all tumors are composed of a heterogeneous cell population, making them difficult to treat. A small cancer stem cell population with a low proliferation rate and a high tumorigenic potential is thought to be responsible for cancer development, metastasis and resistance to therapy. Stem cells were reported to be involved in both normal development and carcinogenesis, some molecular mechanisms being common in both processes. No less controversial, stem cells are considered to be important in treatment of malignant diseases both as targets and drug carriers. The efforts to understand the role of different signalling in cancer stem cells requires in depth knowledge about the mechanisms that control their self-renewal, differentiation and malignant potential. The aim of this paper is to discuss insights into cancer stem cells historical background and to provide a brief review of the new therapeutic strategies for targeting cancer stem cells.

## 1. Introduction

According to the modern theory of carcinogenesis, malignant transformation may occur due to the action of a wide range of mutagenic agents on stem cells present in the adult tissue [1]. The exact origin of cancer stem cells (CSCs) remains unknown, despite intensive research in the last decade. In a study on leukemia, published by Bonnet *et al.* [2] in Nature Medicine in 1997, the existence of a heterogeneous tumor cell population was first mentioned; this cell population was analyzed in terms of proliferation and differentiation. These cells, found in leukemia cell populations, were thought to have stem cells properties, such as self-renewal capacity and high proliferation rate [3]. Another study conducted by Passegué *et al.* [4] demonstrated that in leukemia, the presence of stem cells is necessary and sufficient for maintaining the tumor cell population. It has also been suggested that the unlimited self-renewal capacity of CSCs may be the cause of tumor recurrence [5]. It has recently been demonstrated that CSCs are present in both hematologic malignancies and solid tumors (*i.e.*, breast cancer, brain tumors, malignant melanoma or prostate cancer) [6,7]. Additionally, surface markers of the CSCs have been identified in many types of cancers including: leukemia CD34+/CD38−, breast cancer CD44+/ESA+/CD24−, brain cancer CD133+, multiple myeloma CD138−, pancreatic cancer CD44+/CD24+/ESA+, colon cancer CD133+, liver cancer CD133+ [5], prostate cancer CD44+/CD133+ [6], lung cancer CD133+ and ovarian cancer CD133+/CD44+/CD117+ [7]. After numerous preclinical and clinical studies, it has been shown that adult stem cells could turn into CSCs with specific surface markers [8,9]. Specific targeting of tumor stem cells has been suggested to be a good alternative for cancer treatment [10,11]. Some studies demonstrate that CSCs exist in primary human sarcoma tumors such as bone sarcomas [12], and CD133 has been shown to be a potential marker for identification of the CSCs as seen in a paper by Suva *et al.* [13] where a population of Ewing’s sarcoma family tumor (ESFT) cells expressed CD133 which also fulfilled *in vivo* criteria of CSCs and *in vitro* plasticity properties of mesenchymal stem cells [12,13].

## 2. Tumor Cells *vs.* Tumor Stem Cells

Over the years, a variety of polemical concepts have been generated to explain the process of carcinogenesis. In the early 1900s, scientists first believed that cancer is a somatic cell disorder [14] and soon after Tyzzer, E. introduced the notion of “somatic mutation” in connection with cancer [15]. However, Boveri’s observation [14] was crucial in understanding the process of carcinogens. He believed that chromosomal abnormalities are fundamental to cancer development, anticipating the cancer genetic hypothesis [14]. More convincing arguments and evidence to sustain the cancer genetic hypothesis came from the discovery that chemicals and radiations could act as mutagenic factors [16,17].

The cancer genetic hypothesis was further supported by Knudson’s two-hit theory, postulating that at least two genetic mutations in a tumor suppressor gene are necessary to generate cancer [18]. Two-hit hypothesis of carcinogenesis may explain why people with a family history of cancer do not necessarily develop malignancies. These individuals may inherit a mutated gene, but at least a second mutation is needed for occurrence of cancer. This theory may also explain why people with no family history of cancer can develop cancer, as long as there are at least two genetic mutations that may occur for a variety of reasons [19,20]. In support of the two mutation theory, other clinical observations showed that somatic mutations in the retinoblastoma gene were present in patients with several types of cancer (e.g., sarcomas breast cancer, bladder cancer, lung cancer) [21,22].

In 1976, Nowell, P.C. proposed the multistep genetic model of tumorigenesis [23] and in 2000, Hanahan and Weinberg explained the classical model of molecular transformation in cancer cells [24]. These studies defined the model of carcinogenesis known as the “somatic mutation theory”, stating that cancer is a clonal, cell-based disease, assuming that quiescence is the regular state of cells in the body [24,25].

The “somatic mutation theory” has dominated oncology for more than 40 years; it explains that multistep genetic alteration of recessively acting tumor suppressor genes and dominantly acting oncogenes take place in cells of origin, giving rise to tumor proliferation, invasion, metastasis and drug resistance.

However, the cellular origin of cancer and the mechanisms behind cancer development are still debatable since tumors, be they solid or liquid, are heterogeneous cell populations composed of a large number of tumor and non-tumor cell populations. From this perspective, a new model—the tissue organization field model—tries to explain the development of cancer, meaning that cancer is a tissue-based disease and involves a dynamic communication between the various cell populations coexisting in cancer tissue and also stroma/epithelium interactions [26,27]. These models tried to define the model of carcinogenesis, responsible for both clonal selection and tumor cell heterogeneity.

Recently, in a study by Feinberg *et al.* the epigenetic aspect was added to this theory which accounts for the alterations in global DNA methylation that in turn can induce both abnormal activation of proliferation genes and tumor suppressor genes silencing [28]. In addition, the author suggested that “tumor-progenitor genes” promote epigenetic disruption of stem/progenitor cells and that the epigenetic plasticity together with genetic injuries are responsible for tumor cell heterogeneity and tumor progression [28].

Most of current understanding about the existence of stem/progenitor cells in adult tissue originates from animal model studies [29–31], many authors suggesting that the existence of stem/progenitor cells and the committed progenitors or transit-amplifying cells may enable the malignant transformation [32].

The CSCs theory is an old idea that was first described in 1973 by Moore *et al.* [33]. According to the current CSCs accepted hypothesis, besides the cells that form the tumor bulk, the malignant transformation also involves the existence of a cell population with special properties such as self-healing ability, called cancer stem cells or tumor stem cells [34–36].

CSCs are able to self-heal and sustain tumor growth and heterogeneity. In this context, the CSCs theory has many similarities to the evolution model of whole body. Stem cells that pass through embryogenesis can be normal adult stem cells (tissue stem cell) or differentiated cells. Due to similarities with the evolution model of whole body, it is speculated that an adult stem cell that acquires a genetic mutation, develops into a CSC of origin (CSCO) that acquires several new genetic mutations, developing into a CSC and finally giving rise to cancer [37].

The CSC hypothesis gained credibility because all main cancer-origin theories (genetic/epigenetic events, chemical-, infection-, virus-induced carcinogenesis) indicated that the tissue stem cell is involved in the generation of cancer [2,38–43].

Unlike the traditional theory of carcinogenesis, recent results obtained from different research groups suggest that CSCs are the driving force of tumorigenesis and metastases, and are the cause of tumor properties, such as proliferation, aggressiveness and resistance to treatment [44].

**Figure 1.**
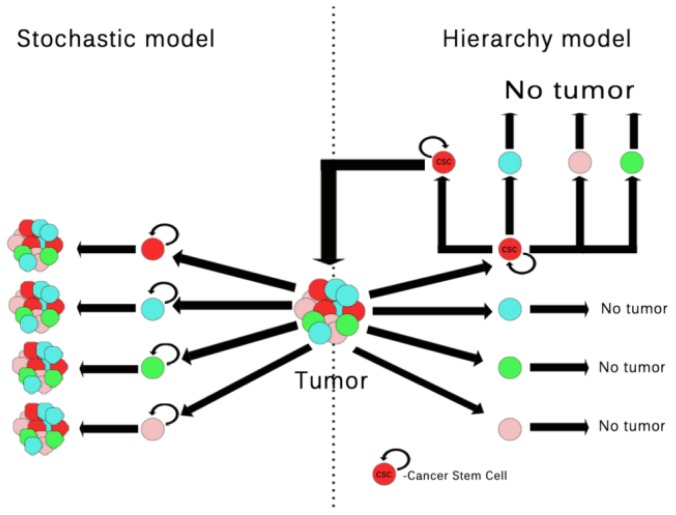
Theory of malignant transformations from adult stem cells. On the left, the stochastic model postulates that several cancer cell types have an equal ability to regenerate and proliferate, each one being capable of giving birth to another tumor (the green, pink, blue and red cells represent similar, non-stem tumoral cell populations); On the right, the hierarchy model postulates that only a specific population (cancer stem cells represented by the red cells marked with a CSC in the middle) has the distinct ability to regenerate, multiply and to differentiate into other subset populations (the green, blue and pink cells), hence giving birth to other tumors.

To explain how cancer occurs and progresses from CSCs, the hierarchy model, also known as clonogenic model, has been proposed. According to the CSC clonal model, only a subset of cancer cells called CSCs are able to initiate malignant progression resulting in heterogeneous tumors (Figure 1) [45–47].

In a study by Chaffer *et al.*, the authors demonstrated that both normal and CSC-like cells can materialize *de novo* from more differentiated cell types. This discovery brings an addition to the hierarchical models that do not take into consideration a special type of cell plasticity in which stem and non-stem states are converted into one another, often as the result of the cell environment [48]. Consequently, researchers have used the stochastic model to illustrate the CSC model of cancer development, whereby particular circumstances in a mix-tumor cell population transform any tumor cell into a stochastically tumor-initiating cell, leading to tumor heterogeneity (Figure 1) [44,49–51].

Tissue organization architecture model and external stimuli were suggested to be important in CSC initiating tumors. In the paper of Vermeulen *et al.*, the authors demonstrated that in colon CSCs, Wnt activity and cancer stemness may be regulated by environmental stimuli. They also showed that by reprogramming, clonogenic differentiated cancer cells can no longer turn into CSCs, and retrieve their tumorigenic capacity when stimulated with myofibroblast-derived factors, suggesting that cancer stemness is not an unyielding characteristic [52].

Roesch *et al.* detected a slowly dividing cell population and demonstrated that it could sustain melanoma growth and self-renew. They also showed that these cells can shift cell state through epigenetic changes mediated by JARID1B, implying cell plasticity [53].

The cancer associated fibroblasts (CAFs) and the epithelial-mesenchymal transition (EMT) have also been reported as important components of the tumor microenvironment induced cell plasticity. The current reports of the CAFs acknowledge that this stimulates cancer cells to express stem cell markers like CD133 or CD44, and stimulate local acidification that in turn alters the extracellular matrix pattern, increasing cancer cell anchorage-independent growth potential and their tumor-repopulating ability [54–56].

EMT is a key factor that is often activated during embryogenesis but also during cancer invasion and metastasis [57,58]. Mani *et al.* [59] showed that “Induction of an EMT in non-tumorigenic and immortalized human mammary epithelial cells results in the acquisition of mesenchymal traits and properties associated with mammary epithelial stem cells”. These findings illustrate a direct link between the EMT and the acquisition of epithelial stem-cell properties [59]. Interestingly, the mechanisms that promote the CAFs and EMT reactivity in cancer cells have been shown to be analogous, both enrolling redox related molecules such as hypoxia inducible factor 1 (HIF) and cyclooxygenase 2 (COX2) [60]. In addition, CAFs were shown to induce EMT by an epigenetic mechanism, which in turn disrupts cancer cells adhesive cell-to-cell interactions, acquiring a mesenchymal motility and escaping from primary neoplastic lesions and metastasis [54,61].

The proposal that CSC are involved in carcinogenesis is consistent with the identification of CSC subpopulation in leukemia [3,4,62], breast cancer [63], prostate cancer [8], ovarian cancer [7] and more recently, in certain types of brain tumors [64].

The percentage of CSCs identified in the whole tumor cell population was not the same in different tumor types [50,65] and the number of the CSCs in the tumor was reported to be correlated with patient prognosis [66,67]. However, other groups showed that tumors of the same histological type contain a very different percentage of CSCs, varying from 0.03% to approximately 100% determined by different techniques involving antibodies against surface markers such as CD133+ or using Hoechst dye [68–71], suggesting that the number of the CSCs is of doubtful prognostic value. Other studies using sphere cultures and differentiation identified CD133+ CSCs in osterosarcoma-stabilized cell line by showing that only CD133+ cells were able to form sarcospheres. Whereas, Aldehyde Dehydrogenase (ALDH) assays are also used to identify populations of CSCs within breast, colon and lung cancer but are also rarely used for identifying osteosarcoma cells [12].

Because CSCs divide slowly, it has also been suggested that the CSC population is responsible for tumor resistance to treatment.

## 3. Molecular Signalling Pathways in Cancer Stem Cells

Little is known about the relationship between CSCs and other heterogeneous cell populations within a certain tumor and even less is known about signalling pathways that these cells use in order to coordinate their behaviour. Considered “The Holy Trinity” of cell molecular signalling, Notch, Hedgehog and Wnt pathways along with the B lymphoma Mo-MLV (Moloney murine leukaemia virus) insertion region 1 homolog polycomb ring finger oncogene known as BMI-1 pathway, are currently the most studied molecules [72].

The Notch pathway plays a major role in both normal and CSCs. Once activated by its ligands (Delta-like and Jagged ligands), Notch receptor translocates to the nucleus and associates with a DNA-bound protein, starting a cascade of transduction events in the cell [73]. In the normal stem cell population, Notch receptor is important in cellular fundamental functions such as proliferation, differentiation and apoptosis. The deregulation of Notch pathway results in abnormal proliferation, reduced differentiation and arrested apoptosis with major implications in a variety of cancers such as: T-cell acute lymphoblastic leukemia, melanoma, breast cancer, meningioma and lung adenocarcinoma [74,75].

Hedgehog is a molecule responsible for a plethora of effects on the early development of different parts of the body. The Hedgehog pathway was first discovered by experimental biologists in the Drosophila Melanogaster fly [76]. After further research, three different homologues were discovered in the human body, each of them having a distinct role: Sonic Hedghehog (SHH), the Desert Hedgehog (DHH) and the Indian Hedgehog (IHH) [76]. Present in both embryo and adult stem cells, the SHH is involved in the development of the Central Nervous System (CNS), limbs and axial skeleton, IHH is involved in cartilage differentiation while DHH is strongly linked to the development of germline cells and Schwann cells [74]. The message from the Hedgehog receptors is downstream transmitted through the messenger molecules. Some proteins, such as Fused or Costal 2, have been reported to be part of the Hedgehog signalling, but most of the signalling proteins are still unknown and further research is required to find the downstream receptors pathway [70]. Hyperactivation of Hedgehog pathway has been linked to several types of cancer such as basal cell carcinomas, advanced prostate cancer [71], advanced gastric adenocarcinomas [74] and medulloblastomas [77].

Another pathway involved in growth, survival and stem cell self-renewal is the *Wingless Drosophila melanogaster* segment-polarity gene and *Integrase-1* vertebrate homologue (Wnt) pathway. The members of the Wnt family consist of secreted lipoprotein ligands. By binding to various receptors, Wnt ligands trigger receptor intracellular signalling, switching off the GSK-3β-dependent degradation pathway, which in turn enables β-catenin to accumulate in the cytosol and to translocate the nucleus to activate transcription of the Wnt target genes. Thus, in the active state, Wnts bind to receptors of the Frizzled and LRP5/6 co-receptors on the cell surface leading to β-catenin protein stabilization and triggering dislocation of the GSK-3β kinase from the APC/Axin/GSK-3β-complex. In the active Wnt signals, β-catenin interplays with CK1 and the APC/Axin/GSK-3β-complex, activating β-TrCP/SKP pathway that induces protein ubiquitination and proteasomal degradation.

The Wnt/β-catenin is a well conserved pathway that regulates stem cell pluripotency during development. Aberrant Wnt signalling underlies a wide range of pathologies in humans, including cancer. Regarding its influence and implication in certain malignancies, the WNT pathway was incriminated to be linked to colorectal cancer, medulloblastoma and lymphoblastic leukemia. In most cases, the common denominator of the abnormal activity of this pathway is gene transcription activation by the protein β-catenin [78,79]. Several studies on genes activated in CSCs show that a large network of transcription factors (TFs) can be activated in these cells that are unlikely to ever occur in normal physiological conditions [80]. Among different CSC TFs, the *Achaete-Scute Homolog 1* (*ASCL1*) gene is considered to be an upstream regulator of the Wnt signalling pathway. In a paper by Rheinbay *et al.* [80], ASCL1 was shown to activate Wnt pathway by repressing the negative regulator *Dickkopf-related Protein 1* (*DKK1*) gene in glioblastoma (GBM) CSCs. It has been recently demonstrated that ASCL1 is crucial for GBM CSCs *in vivo* tumorgenicity [81].

Identified as a member of the Polycomb group, the BMI 1 pathway has been directly linked to self-renewal and differentiation of human stem cells. Its most significant effects have been associated with hematopoiesis, skeleton development and neural growth. BMI 1 deficient mice present an overwhelming depletion of nervous stem cells which translated into progressive post-natal neurological retardation and other significant defects. BMI 1 depletion also results in a significant reduction in self-renewal capacity of the HSCs [82].

BMI 1 over-expression and amplification have also been found in several types of malignancies, most notably in hematological ones such as leukemias or mantle cell lymphomas [83] due to its direct implication in self-renewal of hematopoietic stem cells. Other studies link the BMI 1 pathway to: gliomas [82], nasopharyngeal carcinomas [84] and ovarian cancer [85]. Recently, a link between the Hedgehog pathway and the BMI-1 gene has been found in several types of cancer, such as breast, basal cell carcinomas, medulloblastomas and esophageal cancers [86,87]. It has been suggested that in breast cancer the BMI-1 gene was present in the downstream signalling of the Hedgehog pathway and that activation of the same pathway determines an upregulation of the BMI-1 expression [88].

Cleton-Jensen *et al.* [89] have demonstrated that miRNAs are also implicated in emergence of CSCs from ESFTs. They were able to present a scenario where deregulation of miRNA in CSCs could be the cause of expression of several TFs that help generate and sustain tumorgenicity in CSC populations. Although miRNA repression is a key feature of malignant cells, there is little evidence of shared miRNA profiles between different types of cancer cells, this group considered that because different transcript expressions can be regulated by different miRNAs, it is easy to believe that CSCs derived from different types of tumors could survive by exploiting different miRNAs to regulate expression of their essential TFs. An example could be the ESFT and breast cancer CSCs that have been demonstrated to share the same miRNA profile for *c-Myc*TF regulation [90].

## 4. Targeted Therapy against Cancer Stem Cells (CSCs)

**Figure 2.**
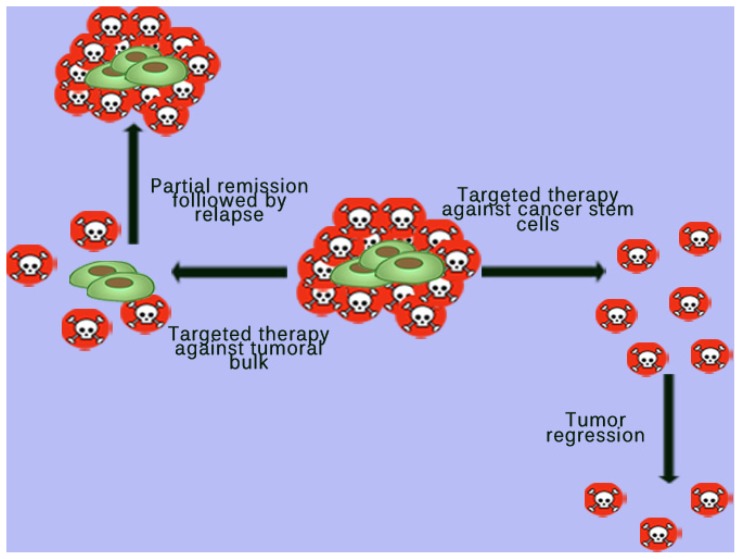
Targeted therapy against cancer stem cells. Therapeutics aimed at the general population of cancer cells has a limited impact on cancer stem cells (CSCs). In time, their regenerative and proliferative capabilities are responsible for the relapse. On the contrary, therapy aimed at the CSC population determines a destruction of the population responsible for differentiation and proliferation which in turn produces a much more pronounced and stable tumoral regression. The CSCs are displayed as the green, larger cells while the non-stem cells are depicted as the smaller, red with the skull and bones in the middle.

Although advances have been achieved in cancer therapy by using modern techniques and therapeutic approaches such as personalized targeted therapy, the solid tumor cells are generally resistant to treatment and patients continue to exhibit a poor prognosis [91]. The concept that tumor development and progression depends on the evolution of CSCs dramatically changes the possibilities in which cancer can be cured. This CSC population is very important in the tumor’s malignant potential and response to therapy [2]. It is known that cancer therapy targeting the tumor cell bulk can produce a partial regression of the tumor, followed by the appearance of new tumor clones, developed from existing CSC population. Therefore, identification and targeting of neoplastic stem cells represents a major challenge in modern cancer therapy and researchers are trying to find new molecular therapies directed specifically against these cells (Figure 2). Thus, molecular therapies against CSCs were reported to be more effective, as they induce tumor regression by reducing the occurrence of new cancer cells (Figure 2) [49,65].

Many studies in clinically relevant cancer therapy are based on the hypothesis that there is a strong relationship between the tumor resistance to conventional therapy and the CSCs intrinsic mechanisms of resistance to conventional chemotherapeutic drugs and radiation therapy. Targeting CSCs or specific pathways responsible for radiation or drug resistance were reported to improve the treatment effect. CSCs harbor numerous intrinsic mechanisms of resistance to conventional chemotherapeutic drugs, radiation therapy, and novel tumor-targeting drugs that permit CSC survival of current cancer therapies and CSC-mediated initiation of tumor recurrence and metastasis [92–96].

In the last years, some insight regarding the intracellular and extracellular mechanisms that regulate stem cell division and differentiation has been provided. Different intrinsic and extrinsic factors were proposed to influence CSC expansion. These factors, present in CSCs, have been divided into the following categories: survival, differentiation, multidrug resistance, signal-transduction and oxidative stress factors [97]. New generation of cancer therapeutic drugs are designed to eliminate CSCs by interfering with the pathways mentioned above. These drugs have been examined *in vivo* and *in vitro*.

It has been speculated that some stem-cell signal pathways such as Wnt, Fibroblast Growth Factor (FGF), Notch, Hedgehog, and TGFβ/BMP (Bone Morphogenetic Protein) signaling play an important role in the pluripotent stem cells and CSCs homeostasis [98]. Using high throughput screening methods, several pharmaceutical companies developed drugs that specifically target these pathways. Inhibition of Wnt/β-catenin signalling pathway by siRNA or by small molecule XAV939 was reported to induce cell death in several types of cancer cells [99,100]. Sonic Hedgehog signalling has also been evaluated as targeted pathway in regulating CSC growth in many cancer types. Some small-molecule modulators of hedgehog signaling have shown success in treating medulloblastoma, basal cell carcinoma, pancreatic cancer and prostate cancer [101]. In addition, blocking the Hedgehog signalling by siRNA chemosensitized the hepatocellular carcinoma cells to 5-fluorouracil (5-FU) treatment [102].

It is known that CSCs can be identified by several cell surface antigens such as CD133, CD90, CD44, OV6, and CD326 (EpCAM). EpCAM is considered an epithelial differentiation marker, however only when associated with stemness markers such as CD133, it can be used as a progenitor CSC marker in experiments.

Studies show that hepatic CSCs can be identified using several markers including CD133, CD24, CD44, CD90 and EpCAM [103]. A study by Chen *et al.* [104] demonstrated that CD133+, EpCAM+ cells, showed similar properties as tumor initiating cells (TICs) in Huh-7 cells, such as high differentiation capacity, increased potential of colony-formation, preferential expression of stem cell-related genes, MDR to some chemotherapeutics, more spheroid formation in cell cultures and higher tumorigenicity in NOD/SCID mice. Another study on pancreatic cancer stem-like cells demonstrated that isolated CD44+, CD133+, EpCAM+ cells of human pancreatic cancer behave as cancer stem-like cells, which show more aggressive behavior such as increased cell growth and migration, clonogenicity, and self-renewal capacity [105].

Thus, antibody-based therapeutic approaches targeting CD133, CD90, CD44, OV6, and CD326 are currently being developed [106,107].

Although a specific antibody may be effective in eradicating CSC, it is important to note that in most situations, this therapeutic method is not enough to eradicate the whole tumor. It has been hypothesized that tumor cells can escape the cytotoxic effect of specific antibodies by decreasing the expression of surface antigen, by developing resistance to cancer chemotherapeutic agents or by acquiring multiple mutations. For this reason, therapeutic approaches targeting CSCs in combination with traditional cancer therapies have been also used in preclinical experiments and in clinical trials [107].

Retinoic acid, a stem cell differentiation agent, has been used in combination chemotherapy in treatment of acute promyelocytic leukemia [108]. Inactivation of intracellular stem cell signalling pathways in combination with self-renewal of CSCs has also been experimented *in vivo* and *in vitro* [109,110].

Hermann and colleagues reported that a combined regime of a new hedgehog pathway inhibitor SIBI-C1 with Rapamycin and Gemcitabine yielded very promising results. The triple treatment completely depleted the CSCs population in primary human pancreatic cancer tissue xenografts having a profound effect on the tumoral stroma as well. In order to assess the effect on these therapeutic agents on normal stem cells, blood levels were followed throughout the study and compared to a standard gemcitabine regime. The triple combination treatment did not yield any significantly elevated side effects as it was initially expected to do [111].

In another study, Guofang Chen *et al.* demonstrated that administration of high levels of metformin in thyroid carcinomas inhibits growth of both thyroid carcinoma cells and CSCs. This effect was suggested to be related to Metformin’s inhibitory effect on the insulin/IGF to 5′ Adenosine monophosphate (AMP)-activated protein kinase (AMPK) and mTOR signalling pathways, which was present in both types of cells. Due to the over-expressed nature of the insulin/IGF and AMPK/mTOR receptors in both stand and CSCs as opposed to normal cells, the inhibitory effects were substantially different in the two populations [112].

A study performed by Chia-Hsin Chan *et al.* has shown that inhibition of the Skp2 protein, a ligase responsible for Akt-mediated aerobic glycolysis, resulted in a reduced self-renewal capacity of CSCs in prostate cancer. Inhibition of Skp2 also increased tumor cell sensitivity to chemotherapeutic agents. The therapeutic agent had shown a significantly higher affinity and consequential destructive effect for cancer cells in comparison to normal cells, where it showed a noticeably smaller effect over the viability of normal cells [113].

Even though chemotherapy is the treatment of choice against most types of cancers, its effectiveness is limited due to multi-drug resistance (MDR) developed by CSCs. There are three major mechanisms proposed in order to explain MDR, but the most investigated it is the efflux of cellular cytotoxic drugs, related to the over-expression of multifunctional efflux transporters, the ATP-binding cassette (ABC) proteins. ABC efflux pumps confer protection to CSCs, shielding them from the adverse effects of chemotherapeutic insult [90,114–116].

Two models have been proposed to explain MDR in CSCs; one suggests that after chemotherapy only ABC expressing CSCs are able to repopulate the tumor with newly formed CSCs or differentiated progenitor cells. The second model proposes that only CSCs that acquire drug resistance survive under the pressure of mutations and give rise to new and more aggressive drug-resistant cell phenotypes [114]. Therefore, chemoresistant CSCs are considered to be the Achilles’ heel of cancer [117]. Some authors have identified two compounds: DECA-14 and rapamycin that selectively target neuroblastoma (NB) CSC, while having little effect on normal stem cells, preventing NB CSC self-renewal both *in vitro* and *in vivo* [5]. However, ongoing research is focusing on the efficacy of targeting multiple key TF that are responsible for CSCs’ stemness [118].

Unfortunately, most of these new therapeutic approaches are not highly specific for CSCs, because the difference between the types of cell populations existing in the tumor is still difficult to find, since several signaling pathways are involved in regulation of cell proliferation of both CSCs and normal adult stem cells. This means that the percentage of abnormalities including certain distinctive features such as over expression of specific genes and/or receptors is still relatively small. Also, due to the nouvelle nature of therapies targeting CSC signalling pathways, many of the studies are still in preclinical phases of trials where the implications of above mentioned treatments on normal stem cells are difficult to assess. In addition, cancers can be triggered by multiple oncogenic mutations or CSC can suffer multiple mutations. For this reason, CSCs targeted therapy may be limited or may even fail and further research studies are needed to find better methods to specifically eliminate the CSC population.

## 5. Conclusions

Although most questions regarding the origin and certain features of CSCs remain unanswered, their existence within tumors is widely accepted. Also, more clinical and experimental data show that curative cancer therapy is effective only when CSC is completely eradicated. Current therapeutic approaches against cancer have been reported to control cell proliferation and tumor growth but they are not able to completely eradicate the tumor cell mass. Remaining cells often metastasize or recur in the same location. Chemo- and radio-therapy on the tumor bulk may lead to a better therapeutic response, but cannot provide a long-term complete remission of tumors. The role of CSCs in diagnosis and therapy of cancer has recently been the subject of intense research. Therefore, improving anti-cancer treatment response requires more accurate identification of the CSCs.
